# Novel Medicinal Mushroom Blend as a Promising Supplement in Integrative Oncology: A Multi-Tiered Study using 4T1 Triple-Negative Mouse Breast Cancer Model

**DOI:** 10.3390/ijms21103479

**Published:** 2020-05-14

**Authors:** Elisa Roda, Fabrizio De Luca, Carmine Di Iorio, Daniela Ratto, Stella Siciliani, Beatrice Ferrari, Filippo Cobelli, Giuseppina Borsci, Erica Cecilia Priori, Silvia Chinosi, Andrea Ronchi, Renato Franco, Raffaele Di Francia, Massimiliano Berretta, Carlo Alessandro Locatelli, Andrej Gregori, Elena Savino, Maria Grazia Bottone, Paola Rossi

**Affiliations:** 1 Department of Biology and Biotechnology “L. Spallanzani”, University of Pavia, 27100 Pavia, Italy; elisa.roda@unipv.it (E.R.); fabrizio.deluca01@universitadipavia.it (F.D.L.); carmine.diiorio01@universitadipavia.it (C.D.I.); daniela.ratto01@universitadipavia.it (D.R.); stella.siciliani01@universitadipavia.it (S.S.); beatrice.ferrari01@universitadipavia.it (B.F.); filippo.cobelli01@universitadipavia.it (F.C.); giuseppina.borsci01@universitadipavia.it (G.B.); ericacecilia.priori01@universitadipavia.it (E.C.P.); mariagrazia.bottone@unipv.it (M.G.B.); 2 Laboratory of Clinical & Experimental Toxicology, Pavia Poison Centre, National Toxicology, Information Centre, Toxicology Unit, ICS Maugeri SpA, IRCCS Pavia, 27100 Pavia, Italy; carlo.locatelli@icsmaugeri.it; 3 Clinica Veterinaria Curti Maria Grazia, 26841 Casalpusterlengo (LO), Italy; silvia.chinosi@yahoo.it; 4 Department of Mental and Physical Health and Preventive Medicine, Pathology Unit, Università della Campania “Luigi Vanvitelli”, 80138 Napoli, Italy; andrea.ronchi@unicampania.it (A.R.); renato.franco@unicampania.it (R.F.); 5 Gruppo Oncologico Ricercatori Italiani, GORI onlus, 33170 Pordenone, Italy; rdifrancia@iapharmagen.org; 6 Department of Medical Oncology, Istituto Nazionale Tumori, Centro di Riferimento Oncologico (CRO), 33081 Aviano (PN), Italy; berrettama@gmail.com; 7 Biotechnical Faculty, University of Ljubljana, Jamnikarjeva ulica 101, Ljubljana, Slovenia; andrej.gregori@zanaravo.com; 8 Department of Earth and Environmental Science, University of Pavia, 27100 Pavia, Italy; elena.savino@unipv.it

**Keywords:** mycotherapy, breast cancer, lung metastasis, in vivo, complementary medicine, Agaricus blazei, Ophiocordyceps sinensis, Ganoderma lucidum, Grifola frondosa, Lentinula edodes

## Abstract

Although medicinal mushroom extracts have been proposed as promising anti-cancer agents, their precise impacts on metastatic breast cancer are still to be clarified. For this purpose, the present study exploited the effect of a novel medicinal mushroom blend, namely Micotherapy U-care, in a 4T1 triple-negative mouse breast cancer model. Mice were orally administered with Micotherapy U-care, consisting of a mixture of *Agaricus blazei, Ophiocordyceps sinensis, Ganoderma lucidum, Grifola frondosa*, and *Lentinula edodes*. The syngeneic tumor-bearing mice were generated by injecting 4T1 cells in both supplemented and non-supplemented mice. After sacrifice 35 days later, specific endpoints and pathological outcomes of the murine pulmonary tissue were evaluated. (i) Histopathological and ultrastructural analysis and (ii) immunohistochemical assessment of TGF-ß1, IL-6 and NOS2, COX2, SOD1 as markers of inflammation and oxidative stress were performed. The QoL was comparatively evaluated. Micotherapy U-care supplementation, starting before 4T1 injection and lasting until the end of the experiment, dramatically reduced the pulmonary metastases density, also triggering a decrease of fibrotic response, and reducing IL-6, NOS, and COX2 expression. SOD1 and TGF-ß1 results were also discussed. These findings support the valuable potential of Micotherapy U-care as adjuvant therapy in the critical management of triple-negative breast cancer.

## 1. Introduction

Breast cancer (BC) is the most frequent cancer among women [1], impacting 2.1 million women each year, also causing the greatest number of cancer-related deaths. In 2018, it is estimated that 627,000 women died from BC; that is approximately 15% of all cancer deaths among women. While BC rates are higher among women in more developed regions, rates are increasing in nearly every region globally [2].

A great deal of progress has been made in the early detection and treatment of BC; thus, the survival has steadily increased in recent years [3], reaching the five-year relative survival rate at about 89% of the cases. However, not all patients benefited from those progresses, since the lack of effective treatments against metastasis is still the major hindrance to survival and quality of life of patients suffering from BC [4]. Approximately 10%–20% of all BCs are triple-negative breast cancer (TNBC), testing negative for estrogen and progesterone receptors and excess human epidermal growth factor receptor 2 (HER2) protein. This tumor phenotype is associated with either high proliferation or metastasis phenomena [5]. TNBC is the most difficult BC subgroup to treat due to its unresponsiveness to current clinical targeted therapies (e.g., hormonal therapy protocols or chemotherapeutics targeting HER2 protein receptors), high rate of recurrence, and poor prognosis. Presently, people suffering from TNBC receive taxane chemotherapies, such as paclitaxel, as their standard care. Nonetheless, the lack of a targeted therapy and the TNBC heterogeneity highlighted the urgent medical need to identify therapeutic targets and develop novel effective medicines capable to overcome drug resistance in this aggressive BC. A great deal of effort is currently devoted to find out whether certain treatments can interfere with cellular and molecular processes fueling TNBC growth, trying to create new medical therapies able to hinder the typical TNBC metastatic pattern (i.e., frequent occurrence of distant metastases, mainly localized in lung, central nervous system, and bones) often associated with poor prognosis [6,7].

One of the most promising sources for potential drug discovery in cancer therapy is compounds of fungal origin, i.e., medicinal mushrooms, which display anti-cancer, onco-immunological, and immuno-modulating activities, also improving quality of life during chemotherapy [8,9]. Medicinal mushrooms have been employed for hundreds of years, mainly in China and Asia, for the treatment of a wide range of diseases. Over the past 60 years, experimental and epidemiological studies focusing on fungi increased exponentially, and in recent years, the use of some medicinal mushrooms has been approved as adjuvant treatment in cancer therapy in Japan and China. Extensive clinical studies demonstrated their safe use as single agents or combined with conventional antiblastic-chemotherapy [10].

“Micotherapy U-care” is a novel medicinal mushroom blend supplement provided by A. V. D Reform s.r.l. (Noceto, Parma, Italy) consisting of a mixture of mycelium and sporophores extracts of five species, including *Agaricus blazei, Ophiocordyceps sinensis, Ganoderma lucidum, Grifola frondosa*, and *Lentinula edodes.* Anti-cancer, onco-immunological, and immuno-modulating properties of these five medicinal mushrooms have been previously described in vitro [11,12] and in preclinical [13] and clinical study [8].

The anti-cancer activities of mushrooms have been linked primarily to the modulation of the innate and cell-mediated immune response by branched polysaccharides (beta-glucans) or polysaccharides-protein complexes. Moreover, mushrooms contain bioactive high and low molecular weight molecules (triterpenes, steroids, polyphenols, alkaloids, etc.) in all the different mushroom stages, from mycelium to primordium, until the developed sporophore. Some of these bioactive compounds shared activity against aberrantly activated signaling pathways in cancer cells, modulating cell proliferation, cell survival, cell invasion, and angiogenesis [9].

*Agaricus blazei* Murrill was discovered about 50 years ago in Brazil, where it is known as the sun mushroom [14,15]. Anti-tumor and immuno-stimulant properties of *A. blazei* were broadly described [16]. In vitro study on estrogen-positive human BC cell line, MCF-7, demonstrated the anti-proliferative and anti-metastatic effect of an isoflavone-conjugated glycoprotein, extracted from *A. blazei* [17]. *A. blazei* revealed anti-oxidant activity in aging rats [18]. A phase I clinical study by Ohno analyzed the safety issues in BC patients in remission [19]. The quality of life (QoL) in gynecological cancer patients undergoing chemotherapy improved with *A.blazei* extract consumption. Specifically, the treatment increased the activity of the natural killer cells and reduced the adverse effects caused by conventional chemotherapy [20].

*Ophiocordyceps synensis* (Berk.), native to high-altitude regions of Himalayas, consists of a fungus that parasitizes the larvae of the ghost moth (*Lepidoptera*), producing a fruiting body used as a Chinese herbal remedy. Several in vivo investigations showed the efficacy of *O. sinensis* in reducing BC metastasis [21]. This anti-cancer effect was also studied in 4T1 tumor-bearing mice growing metastatic breast tumors, describing effects on a panel of cytokines [13]. *O. sinensis* has also been shown to possess immunomodulatory [22] and anti-oxidant [23] properties.

*Ganoderma lucidum (*Ling Zhi, Reishi) is one of the most popular Asian fungi. Triterpenes, ganoderic acid, and their derivatives demonstrated a direct cytotoxic activity on a variety of cancer cells, including BC [24]. An extract containing β-glucans and triterpenes suppressed proliferation and metastatic potential of BC cells through the inhibition of Akt kinase and transcription factors AP-1 and NF-Kb [25,26]. A pilot study conducted on 48 BC patients undergoing endocrine therapy, described an improvement in cancer-related fatigue and QoL, along with less anxiety and depression and without adverse effects [27]. Many bioactive components of *G. lucidum* have shown potent anti-inflammatory effects [9]. Furthermore, it was also demonstrated that *G. lucidum* extracts decreased IL-8, IL-6, MMP-2, iNOS, IL-1, TNF-α, and MMP-9 secretion in human TNBC cells [28].

*Grifola frondosa (*Maitake*, “*dancing mushroom” in Japanese*)* is a culinary mushroom. In vitro studies using MCF7 cells described cellular mechanisms involved in apoptosis induction and neoplastic cell proliferation arrest [29,30]. Other *G. frondosa*–induced cellular mechanisms, such as a low P27 gene expression, have prognostic implications in early stages of mouse mammary pre-neoplasia [31], while a reduced activity of ITGA2 is highly associated with disease progression and clinical outcomes in BC [32,33]. The reduction of oxidative stress, particularly in terms of increased mitochondrial superoxide dismutase 2 (SOD2), was described in vitro [30]. Phase I/II clinical trials in BC patients demonstrated that oral administration of a *G. frondosa* extract is associated with both immunologically stimulatory and inhibitory measurable effects in peripheral blood [34].

*Lentinula edodes (*Shiitake) is an edible mushroom native to East Asia, where it has long been considered a delicacy as well as a medicinal mushroom. In vitro studies using human BC cell lineages exposed to *L. edodes* revealed cell growth inhibition paralleled by apoptosis induction [35]. Interestingly, in vivo investigations, assessing the lung-protective effects of a polysaccharide fraction extracted from *L. edodes* demonstrated a decreased inflammatory pathway activation paralleled by an improved antioxidant status [36]. Moreover, the efficacy of *L. edodes* mycelia extract was tested in BC patients undergoing post-operative adjuvant hormone therapy, demonstrating an improvement of immune function and QoL scores after a long-term oral administration [37]. Similarly, the concomitant use of mycelial *L. edodes* extract and oral immunomodulatory (REC75 therapy) can maintain host QoL and immune function [38]. An epidemiological study evaluating host QoL and immune function in BC patients co-administered with a combination of adjuvant chemotherapy and *L. edodes* mycelia extract supported the application of this extract as a useful oral adjuvant to anthracycline-based chemotherapies [39].

In the present investigation, we exploited the effect of an oral supplementation with “Micotherapy U-care” (M. U-care), a novel medicinal mushroom blend, consisting of a mixture of extracts from the above reported five varieties, in a 4T1 triple-negative mouse BC model. We addressed specific endpoints and pathological outcomes of the murine pulmonary tissue, the lung being one of the distant organs frequently involved in typical metastasis pattern of primary TNBC [7]. We evaluated different markers of general lung toxicity, inflammation, fibrosis, and oxidative stress, both in parenchyma and metastases. In particular, morphological and molecular investigations included (i) pulmonary histopathological and ultrastructural evaluation and (ii) immunohistochemical assessment of pivotal markers of inflammation (i.e., TGF-β1 and IL-6) and oxidative stress (i.e., NOS2, COX2 and SOD1), respectively. Comparative data assessment was analyzed in terms of the biological responses to the Micotherapy U-care blend supplementation, starting before 4T1 injection and lasting until the end of the experiment, concerning either persistence or tendency to resolution of such effects. The QoL was evaluated in a comparative approach.

## 2. Results

**Figure 1:**
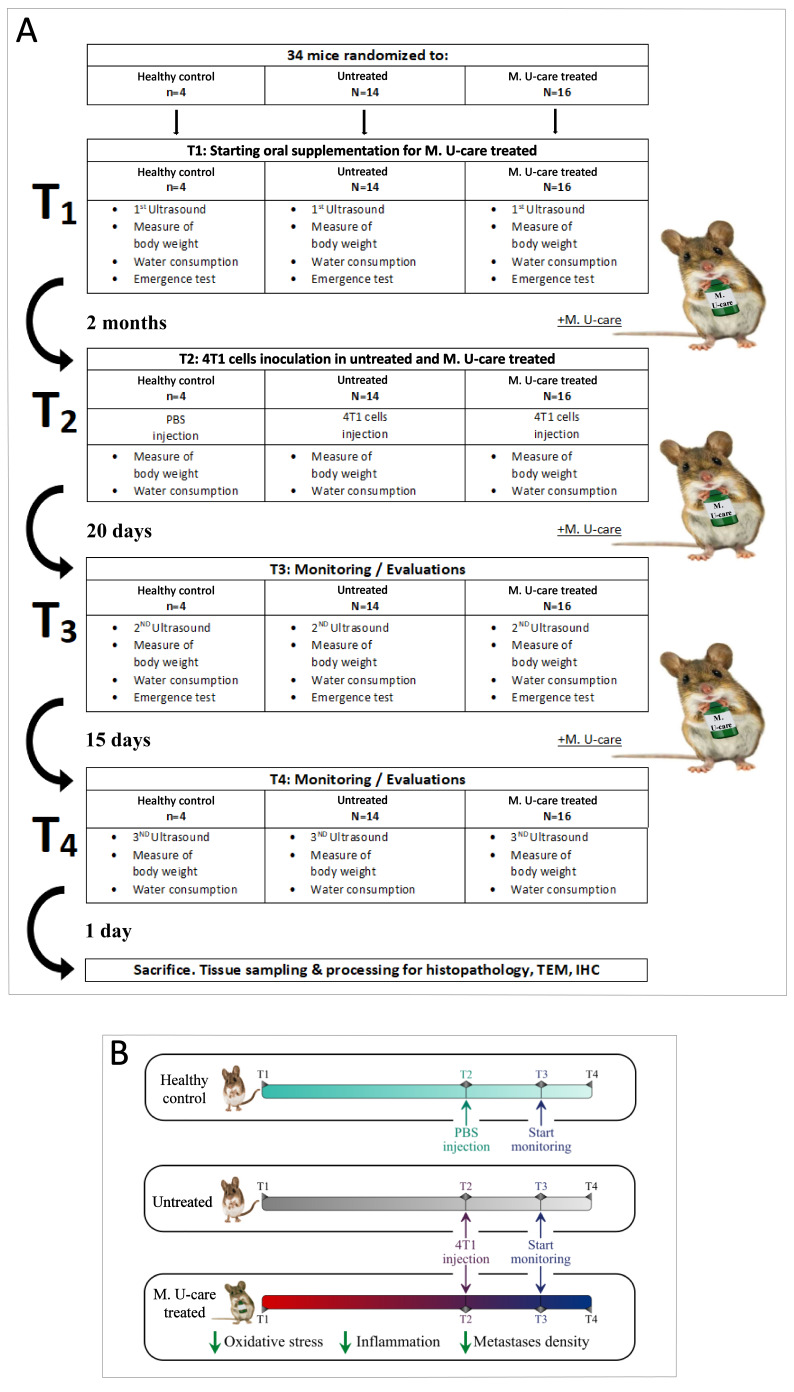
Experimental flow chart (**A**) and schematic drawing highlighting the main outcomes (**B**).

A detailed flow diagram of the experimental design is shown in Figure 1A, together with a schematic drawing (Figure 1B) highlighting the main outcomes of the study.

### 2.1. Quality of Life

First, it has to be underlined that no animals died throughout the whole experimental time course, from T0 to T4; thus, we had a survival rate of 100% in all the three experimental groups.

**Figure 2:**
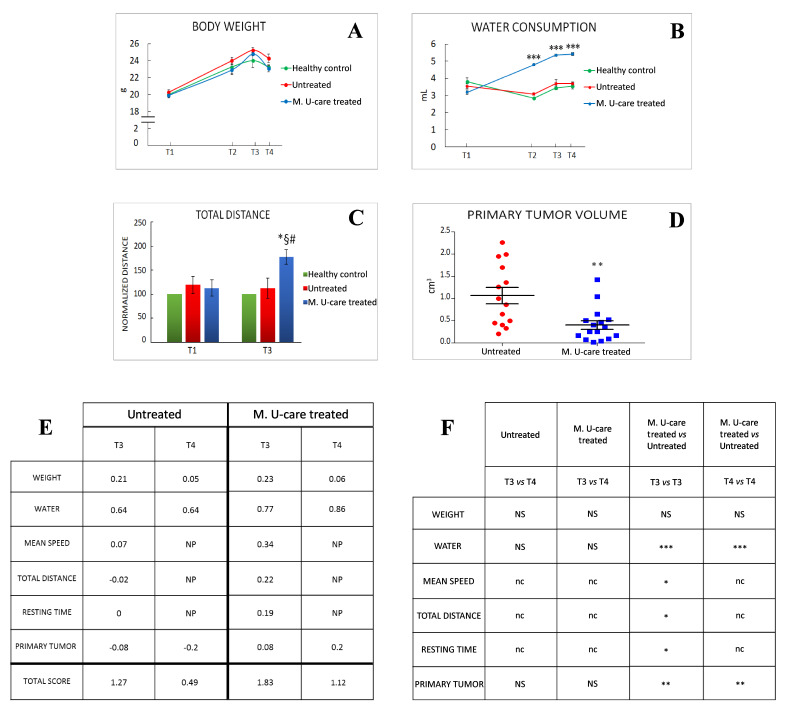
Quality of life and primary tumours features. (**A**, **B**) Weight gain and water consumption, respectively; water consumption: *p* < 0.001 (***) (two-way-ANOVA and Bonferroni’s post-hoc test) was obtained comparing Micotherapy U-care (M. U-Care) treated to all other data reported. (**C**) Normalized value on healthy mice of total distance travelled during the 8 min of emergence test; *p* < 0.05 (*) (two-way-ANOVA and Bonferroni’s post-hoc test) was obtained comparing M. U-Care treated to all other data reported. *: M. U-Care treated mice at T3 *vs* Untreated animals at T3. §: M. U-Care treated mice at T3 *vs* Untreated animals at T1. #: M. U-Care treated mice at T3 *vs* M. U-Care treated mice at T1. (**D**) Primary tumour mass volume: dot plot chart showing the entire mice population (each dot represents an individual mouse); *p*< 0.01 (**) (unpaired Student’s t-test) was obtained comparing Untreated and M. U-Care treated groups. (**E**) Global quality life scoring index; NP: not performed. (**F**) Statistical analysis regarding global quality life scoring index: *p* < 0.01 (**) and *p* < 0.001 (***) (two-way-ANOVA and Bonferroni’s post-hoc test) were obtained comparing M. U-Care and Untreated mice concerning the following parameters: weight, water consumption, and primary tumour; NS: not significant. *p* < 0.05 (*) (unpaired Student’s t-test) was obtained comparing M. U-Care treated and Untreated groups concerning the following parameters: mean speed, total distance, and resting time; nc: not comparable.

We monitored the quality of mice life, before and after 4T1 cells (or PBS) injections. We monitored the quality of mice life in the three experimental groups by creating a composite indicator of physical well-being formed by the body weight (Figure 2A), water (Figure 2B) and food consumption, and locomotor activity (Figure 2C).

Locomotor activity records measured in both M. U-care–treated and untreated mice were normalized to healthy controls data, at each corresponding experimental time (i.e., T1 and T3).

Furthermore, we measured the primary tumor volume. At T4, the primary tumor mass measured in M. U-Care–treated mice was significantly smaller compared to untreated animals (Figure 2D). As the core component of this multidimensional QoL assessment, we evaluated three items based on the locomotor activity determined in behavioral spontaneous test, such as average speed, total distance, and resting time. We checked the selected parameters throughout the entire experimental time (Figure 2E,F).

Notably, the scoring index showed that both at T3 and T4, M. U-care–treated mice reached a higher QoL score index compared to Untreated animals, and that the quality of life decreased in both experimental groups during time (Figure 2E). Nonetheless, the T4 score value measured in M. U-care–treated mice was comparable to the T3 score value determined in untreated animals.

At T4, all mice decreased about 4% of their body weight, this diminution probably being due to the deterioration of their general condition 35 days after the tumour injection. Noticeably, throughout the entire experimental time, the M. U-care–treated mice have the significantly highest water consumption compared to both untreated animals and healthy controls.

### 2.2. Histopathology (H&E Staining) and Ultrastructural Analysis (TEM)

We evaluated (i) the differential injury extent in lung parenchyma and (ii) morphological characteristics of metastases, comparing healthy controls, untreated, and M. U-care–treated mice by Haematoxylin and Eosin (H&E) and transmission electron microscopy (TEM).

**Figure 3:**
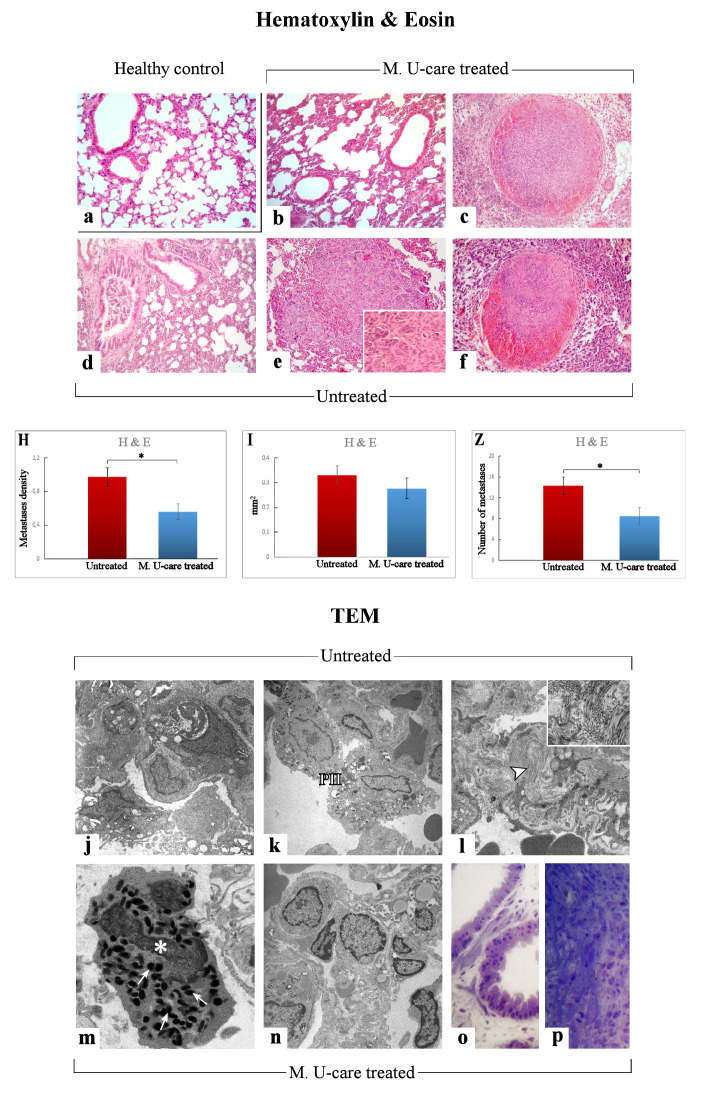
Representative lung parenchyma and metastasis specimens, investigated by both light (H&E) and llectron microscopy—TEM (**a**–**f** and **j**–**p**, respectively), from healthy control (**a**), Mic U-care–treated (**b, c** and **m**–**p**), and untreated (**d**–**f** and **j**–**l**) mice. The physiological pulmonary structure is clearly preserved in healthy controls (**a**). Selected structural alterations evidenced in pulmonary parenchyma as well as metastases feature of both M. U-care–treated and untreated mice are presented. PII: type II pneumocytes; arrowhead: collagen fibril bundles; asterisk: activated granulocyte; thin arrows: cytoplasmic crystalloid-containing eosinophil granules. Light (methylene blue staining on semithin sections) (**o, p**) and electron microscopy details of untreated (j-l) and M. U-care–treated (**m**–**p**) murine lung tissue. Light microscopy magnification: 40× (**a**–**f**); 60× (insert in **e**). Electron Microscopy original magnification: 5000× (**j**–**l, n**); 12,000× (**m**); 20,000× (insert in **l**), 60× (**o, p**). Histograms showing the quantitative analysis of density (**H**), area (**I**), and number (**Z**) of metastases. *p* value calculated by unpaired Student’s t-test: (*) < 0.05.

H&E results demonstrated that the physiological pulmonary structure is clearly well preserved in healthy control mice (Figure 3a), while morphological alterations were manifest in the lung parenchyma of the tumor-induced mice, both M. U-care–treated vs. untreated mice, with the latter showing the more striking injury. Specifically, wall-thickened collapsed alveoli and desquamation, hemorrhagic foci, and parenchymal fibrosis were observed in non-supplemented mice.

In M. U-care–treated mice the metastatic density as well as the number of metastatic nodules were significantly reduced compared to untreated animals (0.56 ± 0.09 vs. 0.97 ± 0.11 and 8.5 ± 1.66 vs. 14.29 ± 1.65, for density and nodules number, respectively). A slight decrease of the single metastatic area was also determined in M. U-care–treated mice (0.28 ± 0.04) compared to jntreated animals (0.33 ± 0.04) (Figure 3H,I,Z and Table 1).

The qualitative observations revealed that the neoformations were widely distributed in the width of the lung, often localized in the outer layers. The metastatic tissues displayed heterogeneous cellularity, with prevalent nodular organization typically characterized by defined, tight clusters of round cells, but often exhibiting several other cell morphologies (e.g., fusiform, cubic, cylindrical shape) and dimensions, with the cytoplasmic part varying greatly. Fewer metastases showed columnar organization with homogeneous cellularity, displaying typical cell ropes with parallel orientation (Figure 3b–f and Table 1).

TEM examination revealed severe modification of the physiological pulmonary structure, characterized by wall-thickened collapsed alveoli and the presence of type II pneumocytes characterized by piles of vacuolated and surfactant-engulfed lamellar bodies (Figure 3 j,k,n–p). An inflammatory condition of the pulmonary tissue (i.e., enhanced presence of activated macrophages, lymphocytes and other inflammatory cells) was also detected together with parenchymal fibrosis, i.e., collagen fibril bundles deposition and accumulation (Figure 3l). These alterations appeared more pronounced in Untreated animals compared to M. U-care treated mice. Notably, the widespread presence of several activated eosinophilic granulocytes, exhibiting the characteristic presence of several cytoplasmic crystalloid-containing eosinophil granules, was evidently increased in M. U-care–treated mice (Figure 3m).

### 2.3. Picrosirius Red Staining: Fibrillar Collagen Networks Evaluation

**Figure 4:**
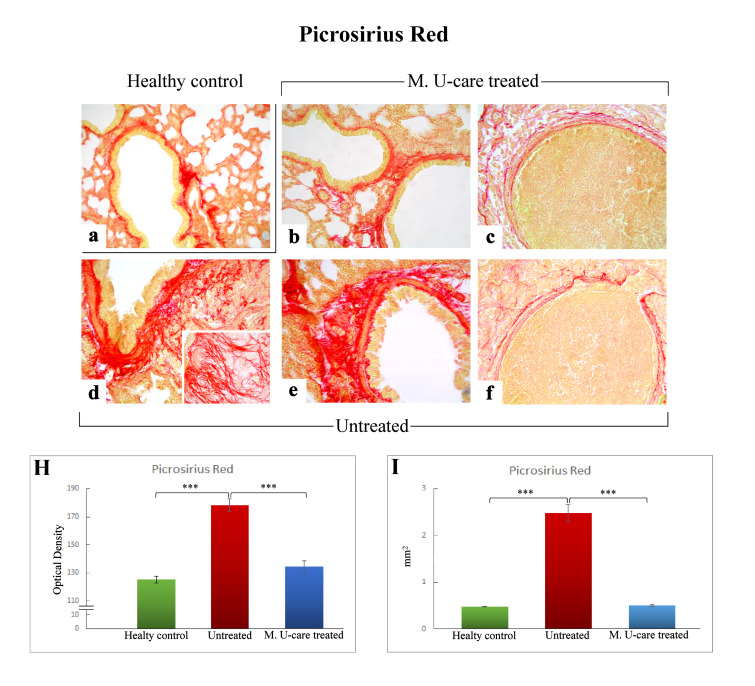
Representative Picrosirius Red stained pulmonary specimens (**a**–**f**) in healthy control (**a**), M. U-care–treated mice (**b**,**c**), and untreated (**d**–**f**) mice. A widespread occurrence of collagen fibrils bundles radiating from bronchiolar wall to the lung parenchyma, also inside the tumours masses, are observable (**b**,**d**,**e**). Collagen fibers encircling the metastases outline are also shown (**c**, **f**). Light microscopy magnification: 40× (**a**–**f**); 60× (insert in **d**). Histograms showing the quantitative analysis of collagen fibers OD (**H**) and bronchiolar wall area (**I**). *p* value calculated using one-way ANOVA followed by Bonferroni’s post-hoc test: (***) < 0.001.

Picrosirius Red staining is one the most sensitive methods to evaluate collagen fibers in paraffin sections [40]. Collagen fibers were predominantly localized in the metastases’ marginal zone, notably encircling their outline. Moreover, several specimens displayed collagen fibrils bundles radiating from the bronchiolar wall to the lung parenchyma, also inside the tumors masses (Figure 4b,d,e).

The successive quantitative analysis evidenced an extremely significant enhancement of the histochemically positive bronchiolar wall area in untreated mice (2.48 ± 0.18 mm^2^), compared to both M. U-care–treated animals and healthy controls (0.51 ± 0.03 and 0.48 ± 0.01 mm^2^, respectively; Figure 4I). Similarly, concerning the bronchiolar wall OD, an extremely significant increase of the collagen fibers optical density was detected in intreated mice (178.03 ± 4.51) compared to both M. U-care–treated animals and healthy controls (134.48 ± 4.09 and 125.1 ± 2.46, respectively; Figure 4 Panel H).

Notably, concerning both bronchiolar wall area and OD, the comparison between M. U-care–treated mice and healthy controls showed the absence of any statistically significant difference, revealing a similar trend in these animal groups (Figure 4H,I).

### 2.4. Inflammatory Pathway: IL-6 and TGF-β1 Immunohistochemical Assessment

Based on literature evidences suggesting the key role played by pro- and anti-inflammatory cytokines balance in promoting BCs development, in the present investigation we performed an immunohistochemical evaluation of the presence/distribution of Interleukin-6 (IL-6) and Transforming Growth Factor-beta1 (TGF-β1), as specific markers of inflammatory pathway.

**Figure 5:**
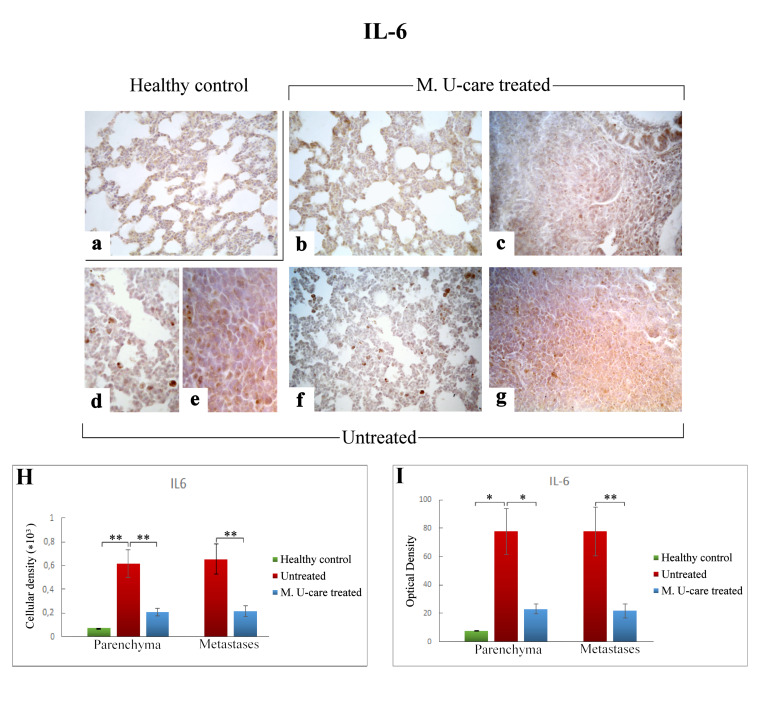
Immunohistochemical labelling for IL-6 in healthy control (**a**), Mic U-care–treated (**b**, **c**), and untreated (**d**–**g**) mice. While a weaker immunoreactivity is shown in M. U-care–treated mice, the heaviest IL-6-immunopositivity is observable in untreated mice in alveolar pneumocytes, in metastatic cells as well as in bronchiolar epithelial cells (**b**–**g**), evidenced by the presence of several immunopositive endothelial cells (**d**,**f**). IL-6 antigen appeared particularly overexpressed in metastatic tissue (**e**,**g**). Light microscopy magnification: 40× (**a**–**c**,**f**,**g**); 60× (**d**,**e**). Histograms showing the quantitative analysis of immunopositive cell density (**H**) and OD (**I**). *p* values calculated by one-way ANOVA followed by Bonferroni’s post-hoc test and unpaired Student’s t-test for parenchyma and metastases, respectively: (*) < 0.05; (**) < 0.01.

**Figure 6:**
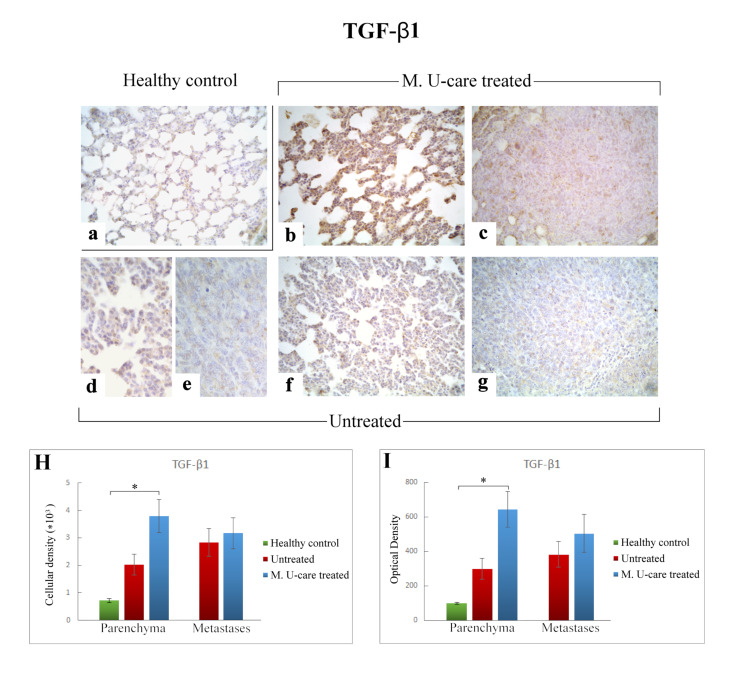
Immunostaining pattern of TGF-β1 expression in healthy control (**a**), M. U-care–treated (**b**,**c**), and untreated (**d**–**g**) mice. The heaviest immunoreactivity is observable in M. U-care–treated mice, mainly localized in alveolar pneumocytes, bronchiolar epithelial cells as well as in metastatic tissue (**b**,**c**). A slight immunopositivity is evident in untreated animals, both at parenchymal and metastatic level (**d**–**g**). Light microscopy magnification: 40× (**a**–**c**,**f**,**g**); 60× (**d**,**e**). Histograms illustrating the quantitative measurement of immunopositive cell density (**H**) and optical density (**I**). *p* values calculated by one-way ANOVA followed by Bonferroni’s post-hoc test and unpaired Student’s t-test for parenchyma and metastases, respectively: (*) < 0.05.

The cellular localization and distribution of IL-6 and TGF-β1, both involved in tissue injury and repair pathways, revealed an extensive spreading in alveolar pneumocytes (both type I and II), in metastatic cells as well as in bronchiolar epithelial cells (Figure 5b–g and Figure 6b–g), particularly evident in collapsed alveolar area, evidencing the cellular inflammatory response.

Regarding lung parenchyma, the heaviest IL-6-immunopositivity was observed in untreated mice at alveolar and stromal levels, with several immunopositive endothelial cells in bronchiolar areas; IL-6 antigen resulted also overexpressed in metastatic tissue (Figure 5e,g). It should be noted that a dramatic increase of IL-6-immunoreactive cellular density (Figure 5 Panel H) and OD (Figure 5 Panel I) were measured in untreated mice (619.55 ± 115.97 and 77.78 ± 16.34, 655.02 ± 125.60 and 77.73 ± 17.04, for parenchyma and metastases, respectively), compared to M. U-care–treated animals (206.33 ± 31.03 and 23.02 ± 3.43, 215.46 ± 47.26, and 21.89 ± 4.94, for parenchyma and metastases, respectively). Notably, any statistically significant difference was determined in lung parenchyma when evaluating cellular density and OD comparing M. U-care–treated mice and healthy controls (206.33 ± 31.03 and 23.02 ± 3.43 vs. 72.23 ± 0.89 and 7.77± 0.14, respectively; Figure 5 Panel H and I).

Differently, the quantitative analysis documented a significant increase of TGF-ß1-immunoreactive cell density (Figure 6 Panel H) and OD (Figure 6 Panel I) in lung parenchyma of M. U-care–treated mice compared to healthy controls (3797.22 ± 598.20 and 645.61 ± 104.41 vs. 714.06 ± 69.04 and 98.31 ± 6.63, respectively). A slight increase of both TGF-ß1-immunoreactive cell density and OD was observed in lung parenchyma of M. U-care–treated mice compared to untreated animals, even though no statistically significant difference was measured (3797.22 ± 598.20 and 645.61 ± 104.41 vs. 2014.33 ± 380.74 and 297.99 ± 60.25, respectively). Likewise, in metastases, a similar trend was observed for both cellular density and OD comparing M. U-care–treated mice and untreated animals (3173.90 ± 567.24 and 504.59 ± 110.11 vs. 2836.22 ± 498.32 and 382.49 ± 75.21; Figure 6H,I).

### 2.5. Oxidative Stress Pathway: SOD1, NOS2, and COX2 Immunohistochemical Assessment

**Figure 7:**
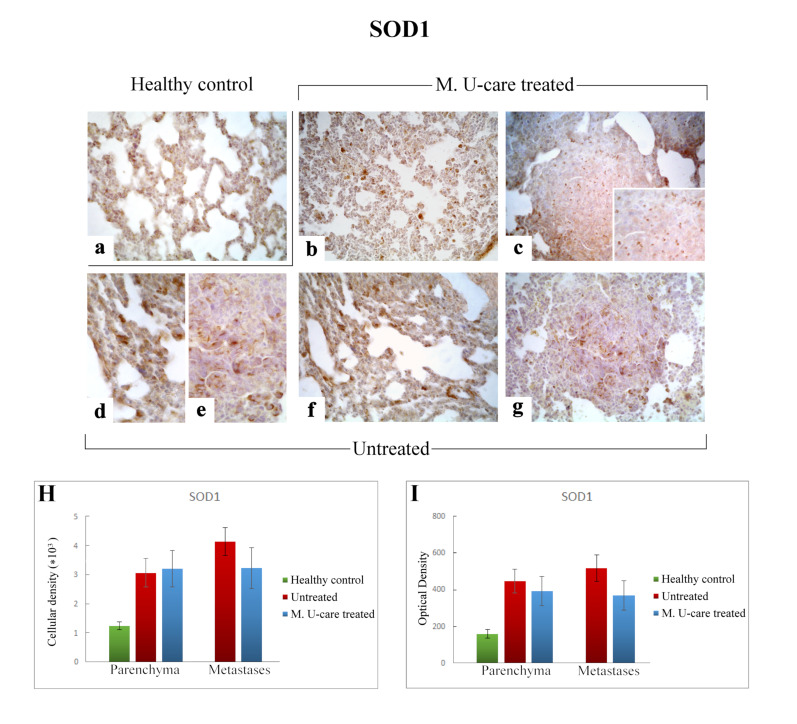
Immunostaining pattern of SOD1 expression in healthy control (**a**), M. U-care–treated (**b**,**c**), and untreated (**d**–**g**) mice. A slightly more marked SOD1-immunoreactivity is observable in untreated animals (**d**–**g**), mainly detectable in alveolar pneumocytes, bronchiolar epithelial cells (**d**,**f**) and in metastatic tissue, also (**e**,**g**). M. U-care–treated mice display a weaker immunolabelling (**b**,**c**). Light microscopy magnification: 40× (**a**–**c**,**f**,**g**); 60× (**d,e**, insert in **c**). Histograms showing the quantitative analysis of immunoreactive cell density (**H**) and optical density (**I**), both in the parenchyma and metastatic tissue. *p* values calculated by one-way ANOVA followed by Bonferroni’s post-hoc test and unpaired Student’s t-test for parenchyma and metastases, respectively.

**Figure 8:**
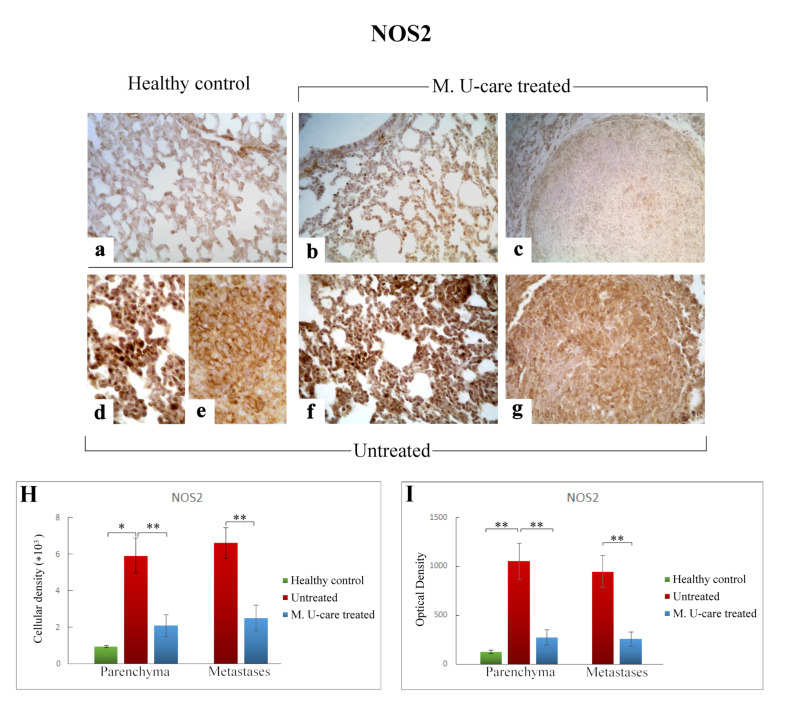
Representative micrographs showing NOS2 expression, detected by immunohistochemistry, in healthy control (**a**), M. U-care–treated (**b**,**c**), and untreated (**d**–**g**) mice. An intensely marked NOS2-immunolabelling is clearly observable in untreated mice (**d**–**g**), with the strongest immunopositivity evident at bronchiolar and alveolar level (**d,f**) as well as in metastases (**e**,**g**). A weaker immunoreactivity is perceivable in M. U-care treated animals (**b**, **c**). Light microscopy magnification: 40× (**a**–**c**,**f**,**g**); 60× (**d**,**e**). Histograms illustrating of the quantitative analysis of immunopositive cell density (**H**) and optical density (**I**). *p* values calculated by one-way ANOVA followed by Bonferroni’s post-hoc test and unpaired Student’s t-test for parenchyma and metastases, respectively: (*) < 0.05; (**) < 0.01.

**Figure 9:**
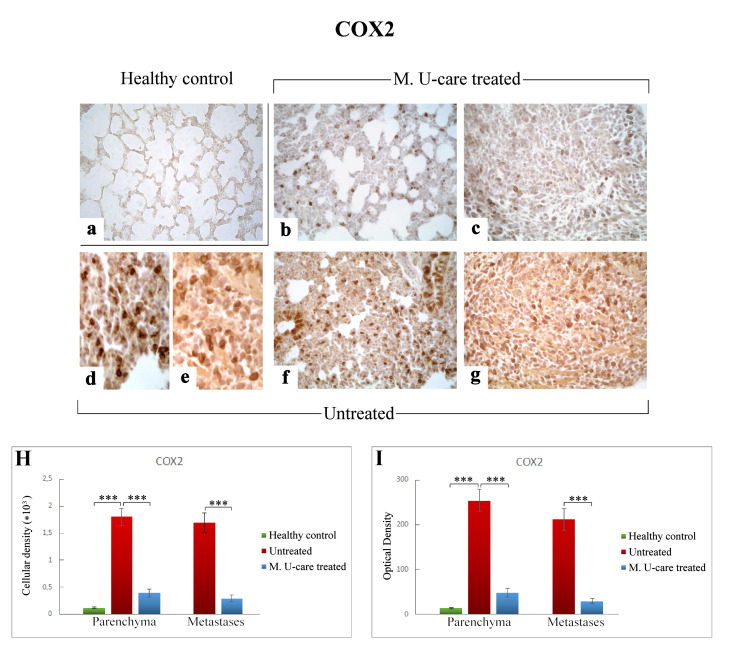
COX2-immunostaining reaction in healthy control (**a**), M. U-care–treated (**b**,**c**), and untreated (**d**–**g**) mice. An extremely marked immunoreactivity is clearly observable in Untreated mice, mainly localized in alveolar pneumocytes and in some bronchiolar epithelial cells (**d**,**f**) as well as in metastatic tissue (**e**,**g**). M. U-care–treated animals display a weaker immunoreactivity (**b**,**c**). Light microscopy magnification: 40× (**a**–**c**,**f**,**g**); 60× (**d**,**e**). Histograms showing the quantitative measurement of immunopositive cell density (**H**) and optical density (**I**), both in the parenchyma and in metastatic tissue. *p* values calculated by one-way ANOVA followed by Bonferroni’s post-hoc test and unpaired Student’s t-test for parenchyma and metastases, respectively: (***) < 0.001.

In the present study, we assessed the presence/distribution of superoxide dismutase 1 (SOD1), nitric oxide synthase 2 (NOS2), and cyclooxygenase 2 (COX2), as specific markers essentially involved in oxidative stress pathway. The localization and distribution of SOD1, NOS2, and COX2, revealed a widespread broadening in bronchiolar and alveolar cells, as well as in the metastatic masses, evidencing the pulmonary reaction to the cancer injury (Figure 7, Figure 8 and Figure 9).

Specifically, the localization of SOD1 showed a widespread distribution in alveolar pneumocytes, in metastatic tissue and some bronchiolar epithelial cells (Figure 7b–g). Noticeably, numerous SOD1-immunopositive activated macrophages were detected particularly evident in collapsed areas, appearing heavily labeled.

SOD1-immunoreactivity evaluated in pulmonary parenchyma in terms of cellular density and OD appeared enhanced in Untreated and M. U-care–treated mice (3068.49 ± 490.54 and 448.33 ± 65.14 vs. 3203.68 ± 627.56 and 392.94 ± 81.70, respectively), compared to healthy controls (1234.90 ± 133.77 and 160.74 ± 21.78), even if these differences were not statistically significant (Figure 7H,I). Concerning the metastatic tissue, similar cellular density and OD values were detected comparing M. U-care treated and Untreated mice (3238.79 ± 702.67 and 368.82 ± 81.03 vs. 4143.29 ± 483.66 and 517.84 ± 28.21, respectively; Figure 7H,I).

Similarly to the observed IL-6 trend, NOS2-immunoreactivity in lung parenchyma, evaluated in terms of both cellular density and OD, significantly enhanced in Untreated mice (5915.47 ± 971.84 and 1051.52 ± 184.63) compared to both M. U-care–treated and healthy controls (2112.37 ± 589.46 and 271.27 ± 76.89 vs. 935.80 ± 72.42 and 124.06 ± 19.29, respectively). Any significant difference was determined when comparing M. U-care–treated mice to healthy controls (Figure 8H,I). The strongest NOS2-immunopositivity was detected at the bronchiolar, alveolar, and metastatic levels in untreated mice, associated with the presence of several immunopositive inflammatory cells (Figure 8d–g). In metastases, a dramatic increase of NOS2-immunoreactive cell density and OD was measured in untreated animals compared to M. U-care–treated mice (6606.67 ± 855.87 and 945.89 ± 160.91 vs. 2474.37 ± 708.35 and 255.57 ± 72.03, respectively; Figure 8H,I).

Accordingly to the above reported data regarding both IL-6 and NOS2, COX2 immunostaining in lung parenchyma evaluated in terms of both cellular density and OD was significantly increased in intreated compared to M. U-care–treated mice (1806.66 ± 159.56 and 253.54 ± 25.38 vs. 394.39 ± 74.68 and 48.48 ± 9.53, respectively). No significant difference was measured when comparing M. U-care–treated mice to healthy controls (394.39 ± 74.68 and 48.48 ± 9.53 vs. 120.52 ± 8.16 and 13.90 ± 1.27, respectively) (Figure 9H,I). In detail, most marked COX2-immunopositivity was mainly localized at alveolar and bronchiolar level, as well as in metastatic areas, often accompanied by the presence of immunoreactive inflammatory cells (Figure 9d–g).

Concerning the metastases, an extremely significant increase of both COX2-immunoreactive cell density and OD was determined in untreated mice compared to M. U-care–treated animals (1700.29 ± 178.33 and 212.04 ± 24.60 vs. 292.15 ± 56.24 and 29.68 ± 5.49, respectively; Figure 9 Panel H and I).

## 3. Discussion

After traditional and novel therapies are widely employed [41], complementary and integrative medicine (CIM) was recently adopted as an innovative approach in oncological care, often associated with positive impacts in cancer patients in terms of better response to treatment, adverse side effects reduction, and quality of life (QoL) improvement [9].

One of the most promising integrative approaches in cancer therapy is mycotherapy, which appears to have several benefits, in terms of improvement of the patients’ overall response rate to conventional oncological treatment, immunity function enhancement, and adverse side effects reduction.

Thus, aiming to explore the potential contribution of mycotherapy in the critical management of cancer patients, in the present investigation, we exploited the effect of a novel medicinal mushroom blend, namely Micotherapy U-care, in a 4T1 triple-negative mouse BC model. Specifically, we studied the murine pulmonary tissue with the goal to deeply comprehend Micotherapy U-care’s impact on typical metastatic pattern of primary TNBC, evaluating the potential effects of its employment as a preventive treatment and as long-lasting supplementation.

Concerning QoL and the composite indicator of physical well-being assessment, it has to be noticed that, throughout the entire experimental duration, the M. U-care–treated mice had the higher water consumption compared to both untreated animals and healthy controls. In accordance to an increasing body of literature, we may hypothesise a role for fibroblast growth factor 21 (FGF21), known to stimulates water-drinking behaviour in mice [42], whose expression is regulated by nutritional status; in fact, changes in the FGF21 level are important for adaption to changes of the nutritional balance such as oversupply of macronutrients or changes of amino acid composition [43,44]. In the present investigation, M. U-care–treated mice only received the oral supplementation with the novel medicinal mushroom blend, whose intake may induce an increase of FGF21 levels, which may have a pivotal role in the thirst response.

H&E staining, recognizing various tissue types and the morphologic changes that form the basis of contemporary cancer diagnosis [45], together with TEM analysis, were used to reveal possible pulmonary morphological and ultrastructural alterations. These changes are dramatically manifest in the lung parenchyma of the tumor-induced mice, with the Untreated animals showing more striking injury regarding wall-thickened collapsed alveoli, hemorrhagic foci and parenchymal fibrosis compared to M. U-care–treated mice demonstrating a milder damage. Notably, concerning the metastatic density, we showed a statistically significant reduction (about 50%) in M. U-care–treated mice compared to untreated animals. Neoformations were often localized in the outer pulmonary area, and displayed heterogeneous cellularity, with prevalent nodular organization typically characterized by defined, tight clusters of round cells, nonetheless paralleled by a sporadic columnar organization with homogeneous cellularity, i.e., cell ropes with parallel orientations [46,47].

Furthermore, an enhanced presence of type II pneumocytes, characterized by piles of vacuolated and surfactant-engulfed lamellar bodies, was also observed in untreated mice. Notably, the widespread presence of several activated eosinophilic granulocytes, exhibiting the characteristic presence of numerous cytoplasmic crystalloid-containing eosinophil granules was evidently increased in M. U-care–treated mice only, thus suggesting that the medicinal mushroom blend may act on diverse cytokines balance, improving immune surveillance [9].

Collagen detection in histological samples represents an important procedure to estimate tissue localization and quantitative expression of connective fibers. In pathological conditions such as fibrosis, which results from an imbalance between collagen deposition and reabsorption due to chronic inflammatory processes, tissue collagen quantification represents an important tool in the clinical diagnosis as well as for outcome prediction and therapy individualization, as in the case of lungs [40]. In this view, Picrosirius Red staining revealed collagen fibrils bundles radiating from bronchiolar wall to the lung parenchyma, also inside the tumor masses; peculiarly, collagen fibers encircled the metastases outline. An extremely significant enhancement of the histochemically positive bronchiolar wall area and OD was measured in untreated mice (about five-fold greater), compared to M. U-care–treated animals. Notably, the comparison between M. U-care–treated mice and healthy controls revealed the absence of any statistically significant difference.

Inflammation is a critical component of tumor progression, invasiveness and metastatic process [48,49,50], specifically in the case of TNBC characterized by high aggressiveness [51].

IL-6 plays a pivotal role in linking chronic inflammation to cancer initiation, growth and metastasis [52,53,54,55]. In the present investigation, a significant decrease of IL-6-immunoreactive cell density was measured in lung parenchyma and metastases of M. U-care–treated mice (about 3–4 fold lower) compared to untreated animals. Interestingly, no statistical difference was determined when comparing M. U-care–treated mice and healthy controls.

We may hypothesize that the lower expression of IL-6 determined in M. U-care–treated mice would be related to a decrease in cancer cell proliferation, in which IL-6 could act on Bcl-2 expression, altering the proliferation/apoptosis balance toward neoplastic cell apoptosis. This hypothesis seems to be strongly corroborated by previous in vitro findings, using BC cell models exposed to *G. frondosa* and *G. lucidum* [9,12,30,56].

Concerning TGF-β, this pleiotropic cytokine regulates numerous biological processes of various tissues in an autocrine and paracrine manner. The role of TGF-β in cancer is complicated, and its aberrant signaling activity is well known to play dual roles in cancer depending on tumor stage and cellular context [57,58,59,60]. Our data demonstrated a significant enhancement of both TGF-ß1-immunoreactive cell density and OD (about 5–6 fold higher) in lung parenchyma of M. U-care–treated mice compared to healthy controls. Diversely, a similar TGFβ1 expression pattern was observed both in lung parenchyma as well as in metastatic tissue, comparing intreated and M. U-care treated–mice, revealing no statistically significant difference concerning cellular density or OD.

Growth factors, cytokines, and enzymes (e.g., TGF-β, IL-6, NOS2, and COX2) are known to stimulate the free radical species (reactive oxygen, ROS, and nitrogen, NOS, species) production, which promote tumor development and progression [61,62]. As a matter of fact, cancer cells express increased levels of anti-oxidant molecules to detoxify from ROS, but such an increase is not enough to counterbalance the intracellular ROS levels [63]. Indeed, a vicious circle takes place in which ROS activates inflammatory cells, stimulating the release of a variety of inflammatory cytokines, which subsequently mediates the tumor niche thus promoting cancer stem cell maintenance, replenishment and switch from epithelial to mesenchymal cell [64].

Literature data reported SOD1 overexpression in malignant BC cells, and the development of novel drug complexes targeting SOD activity can be considered as a promising strategy in the chemotherapy of malignant tumors [65]. In the present study, a similar SOD1 immunoreactivity was measured both in pulmonary parenchyma and metastases comparing M. U-care–treated and untreated animals, suggesting that Micotherapy U-care blend supplementation before tumor cells injection and throughout the following experimental time, seemed to not affect SOD1 expression pattern during tumor progression, until death.

Previous data suggested that co-expression of NOS2 and COX2 is a strong prognostic indicator in TNBC patients [66], also contributing to tumor aggressiveness and poor patient prognosis [67].

It has been widely documented that nitric oxide (NO) functional roles comprise a complex spectrum in the tumor context, touching on all hallmarks of cancer, and being also considered a tumor cell cytotoxic molecule, triggering apoptosis and necrosis [68]. NOS2 expression in cancer cells often predicts poor patient outcome [69].

COX2 is frequently expressed in many types of cancers exerting a pleiotropic and multifaceted role in genesis or promotion of carcinogenesis and cancer cell resistance to chemo- and radiotherapy [70].

In the present investigation, NOS2- and COX2-immunopositivity significantly enhanced in untreated mice, compared to both M. U-care–treated and healthy controls, in terms of cellular density and OD. The strongest immunoreactivity was localized at parenchymal and metastatic level, associated with the presence of several immunopositive inflammatory cells. Regarding the metastases, an extremely significant increase of both NOS2- and COX2-immunoreactive cell density and OD was determined in untreated mice compared to M. U-care–treated animals.

Considering all the molecular markers immunohistochemically investigated, it was noticed that the comparative assessment of lung parenchyma and metastases revealed a matching trend of protein expression. This finding strongly suggests that the tumor microenvironment, consisting of surrounding lung parenchyma, mirrors the cellular processes occurring in metastatic tissue. In fact, our data highlighted that both considered tissue districts exhibited a similar alteration leaning, with the only exception of TGFβ1.

In particular, concerning this latter molecule, we may suppose that the significant enhancement detected in M. U-care–treated mice lung parenchyma only, might be due to the well-known TGFβ1 dichotomous effect [60,71]. Specifically, in the pulmonary normal and premalignant epithelial cancer cells, the increased activation of TGFβ signaling could promote cell-cycle arrest and apoptosis to sustain tissue homeostasis and to suppress aberrant cell growth, thereby playing a tumor-suppressive role. Relevantly, this hypothesis properly fits with the well-known anti-cancer and immuno-modulating activity broadly demonstrated for several medicinal mushroom supplementations.

Ultimately, our present data clearly demonstrated a significant reduced expression of specific oxidative stress markers in M. U-care–treated mice compared to untreated animals. In line with the above hypothesised role of FGF21 in thirst response, it has to be taken into account that this molecule is a potent hormone modulator able to reduce oxidative stress and in parallel increasing autophagy [72]. Concerning autophagy, a major process in maintaining intracellular homeostasis, degrading intracellular waste particularly in damaged tissues [73], it is a known target of FGF21 and a modulator of apoptosis and oxidative stress, but our analyses regarding cell death pathways are still ongoing. Nonetheless, we may hypothesise the activation of a cellular cascade in which an increase in FGF21 level enhances the autophagic occurrence, which may play an anti-metastatic role [73], thus triggering an impediment to tumour progression. This could be link with the dramatic reduced pulmonary metastasis density and nodules number that we detected in M. U-care–treated mice only, compared to untreated animals.

## 4. Materials and Methods

### 4.1. Blend Supplementation

The novel medicinal mushroom blend supplement Micotherapy U-care was provided by A.V.D. Reform s.r.l. (Noceto, Parma, Italy), consisting of a mixture of five fungal species (Table 2).

The identity of fungal strains used in Micotherapy U-care supplement production was confirmed by sequencing ITS regions. Fungal samples, grown in Petri dishes were homogenized using ultra turax homogenizer. Genomic DNA was extracted using DNeasy mini plant kit (Qiagen NV, Venlo, Netherlands) according to the manufacturer’s protocol. ITS regions were amplified using two sets of primers: ITS F 5’-AGAAAGTCGTAACAAGGTTTCCGTAG-3’, ITS R 5’-TTTTCCTCCGCTCATTGATATGCTT-3’, ITS-g F 5’-TCCGTAGGTGAACCTGCGG-3’, and ITS-g R 5’-TCCTCCGCTTATTGATATGC-3’. Amplicons were checked using agarose gel electrophoresis and purified using QIAquick PCR Purification Kit (Qiagen NV, the Netherlands). Purified PCR products were sequenced by Eurofins Genomics (Konstanz, Germany). Obtained sequences from both primer-pair products were joined into consensus sequence and aligned against known DNA sequences using NCBI Nucleotide Blast software.

All strains were maintained in the Mycomedicad.o.o. (Mycomedica Ltd., Podkoren 72, 4280, Kranjska Gora, Slovenia), except for *Agaricus blazei*, which was maintained in the Mycelia BVBA (Veldeken 27 9850, Deinze, Belgium).

Fruiting bodies and/or mycelia were cultivated on organic, plant-based substrate for two to four months at 23 °C in a 1000 ± 100 ppm CO_2_ atmosphere. After harvesting, each kg of fresh material was extracted 3 h at 95 °C with 15 L of water with addition of 10% ethanol. After extraction, solids were filtered out and remaining liquid dried under vacuum (65 °C and 150 mbar) until moisture content lower than 7% was achieved. Dry extracts were milled using a Hosokawa Alpine UPZ160 mill into particles smaller than 200 μm and mixed according to specified proportions (Table 2).

All the raw materials and final products were routinely checked following GMP, in accordance to the hazard analysis and critical control points (HACCP) system, with guaranteed traceability.

In particular, QC of Micotherapy U-care mushroom blend were checked for each batch, as following: the polysaccharide content of Micotherapy U-care was determined using β-Glucan Assay Kit (Megazyme, LTD., Wicklow, Ireland) and expressed as total (α plus β) glucan content and 1,3-1,6 beta-glucans (Table 3).

### 4.2. Cell Culture

The mice BC cell line, 4T1, was obtained from the American Type Culture Collection (ATCC, Manassas, Virginia, USA) and cultured in RPMI-1640 medium supplemented with 10% fetal bovine serum, 1% penicillin/streptomycin at 37 °C in a humidified atmosphere (95% air/5% CO_2_). All cell culture reagents were from Euroclone S.p.A., Celbio S.p.A. (Pero, Milan, Italy).

### 4.3. Animals and Experimental Design

Thirty-four eight-week-old wild-type female mice (strain BALB/c) were purchased from Charles River Italia (Calco, Italy). The pathogen-free mice were acclimatized for at least three weeks before conducting the experiments. Mice were kept in cages with two mice at the Animal Care Facility of the University of Pavia at 21 ± 2 °C, with humidity at 50 ± 10%, and under a 12 h light/dark cycle throughout the experiments. Water and food (standard pellet) were provided ad libitum.

All experimental procedures were performed in compliance with the European Council Directive 2010/63/EU on the care and use of laboratory animals, also following the guidelines set by the institution’s animal welfare committee, the Ethics Committee of Pavia University (Ministry of Health, License number 364/2018-PR, approval date:17 May 2018). Hence, all animals used in this research have been treated humanely according to the institutional guidelines, with due consideration for the alleviation of distress and discomfort.

For experiments, researchers were blinded to the group assignment.

After the acclimatization, for a period of two months, 16 (M. U-care treated mice) out of 34 mice received a drink made of Micotherapy U-care provided by A.V.D. Reform s.r.l. (Noceto, Parma, Italy), consisting of a mixture of mycelium and sporophores extracts of five varieties, including *Agaricus blazei* (20%), *Cordyceps sinensis* (20%), *Ganoderma lucidum* (20%), *Grifola frondosa* (20%), and *Lentinula edodes* (20%).The mycotherapic blend was solubilized in water, in such a way that every mouse received 4 mg of supplement per day. This dose was chosen to mimic the oral supplementation in humans (about 1.5 g/day). The remaining mice, i.e., not-treated (untreated, *n* = 14) and healthy control mice (*n* = 4), were fed without any diet supplementation.

QoL in terms of body weight gain, water, food daily consumption, and locomotor activity were monitored daily.

The syngeneic tumor-bearing mice (untreated and M. U-care–treated) were generated by injecting 10^6^ of the 4T1 cells into the nape of the neck of the female BALB/c mice. The healthy control group was injected with a vehicle of phosphate-buffer saline (PBS). M. U-care–treated mice received Micotherapy U-care until sacrifice. Figure 1 shows the experimental design: T0: animals’ randomization, T1: starting Micotherapy U-care oral supplementation for M. U-care–treated mice, only; T2 (about 2 months later): 4T1 cells inoculation in both untreated and M. U-care–treated mice; T3 (about 20 days later): starting monitoring and evaluations; T4 (about 15 days later): continuing monitoring and evaluations. At selected experimental times (from T1 to T4), stools, hair, and fluids were collected for further analyses. Tumor growth was monitored from T1 to T4 using ultrasound imaging. Mice locomotor activity was monitored at T1 and T3 by using a behavioral test. Primary tumor masses were visible to the naked eye as big nodules. The tumors were measured using a caliper, and the tumor volumes were calculated using the formula V = 1/2 (width^2^ × length), as previously reported [74,75,76]. One day after T4, mice were euthanized.

Blood was withdrawn and centrifuged to obtain serum/plasma for future analysis, and lungs were collected.

Lung preparation for morphophysiological evaluations were performed through vascular perfusion of fixative [77]. After fixation, the lungs were carefully removed (see Section 4.4) and sectioned.

All tissue samples were processed for histopathology (hematoxylin and eosin (H&E) and Picrosirius Red staining), immunohistochemistry and ultrastructural evaluation by Transmission Electron Microscopy (TEM).

### 4.4. Emergence Test

All mice, at selected experimental times (T1 and T3), performed the emergence task, a spontaneous behavioral test, which is a variant of the open-field test that was designed to reduce anxiety by providing a safe enclosure within the open field. The emergence task was used to assess exploratory behavior and locomotor activities. Briefly, the free exploration test consists of housing mice in a compartment prior to giving the animal a free choice between a familiar compartment and a novel one. We carried out emergence tests following previously described protocols [78,79].

Mice activity was quantified by a SMART video tracking system with a selected sampling time of 40 ms/point (2 Biological Instruments, Besozzo, Varese, Italy) and a Sony CCD color video camera (PAL; Sony Europe B.V.- Italian headquarters, Milan, Italy) [79].

### 4.5. The Quality of Life Index

We assessed quality of life (QoL) using six items related to wellbeing and monitoring them during the time. For measuring QoL index, the average value and the standard deviation (SD) for each of the parameters were calculated at starting time (Ts) (T1 for average speed (cm/s), total distance (cm), resting time (s), and primary tumor volume; T2 for body weight (g) and water consumption (ml/die)). The values obtained for each mouse at different times were compared to the average value at Ts using the following formula [79]:

QoL score = [(Value-Mean Value at Ts)/SD at Ts]* ± 0.25(1)

Score values were added and resulted a QoL total score. A higher total score indicates a better quality of life.

### 4.6. Tissue Sampling, Histology, Immunohistochemistry, and Ultrastructural Morphology Evaluations

For each treatment, lung tissues were processed for the following morphological and histochemical evaluations.

#### 4.6.1. Lung Specimens Preparation

At necropsy, the top and the bottom regions of the right lungs of animals belonging to different experimental groups were dissected. Tissue samples were obtained according to a stratified random sampling scheme which is a suggested method for lung tissue in order to compensate for regional differences, which are known to exist in the lung [80] and to reduce the variability of the sampling means.

From each sample, 2–3 blocks were systematically derived, washed in NaCl 0.9% and post-fixed by immersion for 7 h in 4% paraformaldehyde in 0.1 M phosphate buffer (pH 7.4), dehydrated through a graded series of ethanol and finally embedded in Paraplast X-TRA. Eight-μm thick sections were cut in the transversal plane and collected on silane-coated slides.

#### 4.6.2. H&E: Histopathological Observations

Subsequently, to overall evaluate structural changes by light microscopy, H&E staining was performed [81]. The slides were then observed and scored with a bright-field Zeiss Axioscop Plus microscope (Carl Zeiss S.p.A., Milan, Italy). Specifically, five slides (about 20 sections) per animal were analyzed; five microscopic fields were examined in each section for each mouse per time/condition. The images were recorded with an Olympus Camedia C-5050 digital camera and stored on a PC running Olympus software (Olympus Italia, Segrate, MI, Italy).

#### 4.6.3. Picrosirius Red Staining

Serial tissue sections were stained for 1h with a Picrosirius Red solution (0.1% of Sirius Red in saturated aqueous picric acid), followed by a wash in 5% acidified water [82,83], for collagen bundle staining. Finally, the sections were dehydrated in ethanol, cleared in xylene, and mounted in Eukitt (Kindler, Freiburg, Germany).

#### 4.6.4. Immunohistochemistry: Inflammatory and Oxidative Stress Pathways Assessment

Immunocytochemical reactions were carried out simultaneously on slides of different experimental groups to avoid possible staining differences due to small changes in the procedures.

Immunohistochemistry was performed using commercial antibodies on mice lung specimens, to localize presence and distribution of different specific markers indicative of inflammation and oxidative stress: (i) Interleukin-6 (IL-6), (ii) Transforming Growth Factor-beta1 (TGF-β1), (iii) Cu–Zn superoxide dismutase-1 (SOD1), (iv) nitric oxide synthase 2 (NOS2), and (v) cyclo-oxygenase-2 (COX2).

Lung sections of healthy control, untreated, and M. U-care–treated mice were incubated overnight at room temperature in a dark moist chamber with selected monoclonal and polyclonal primary antibodies (Table 4) diluted in PBS. Proper biotinylated secondary antibodies (Table 4) and an avidin biotinylated horseradish peroxidase complex (Vector Laboratories, Burlingame, CA, USA) were used to reveal the sites of antigen/antibody interaction. The 3,3′-diaminobenzidine tetrahydrochloride peroxidase substrate (Sigma, St. Louis, MO, USA) was used as chromogen. The nuclear counterstaining was achieved by employing Carazzi’s haematoxylin.

Then, the sections were dehydrated in ethanol, cleared in xylene, and finally mounted in Eukitt (Kindler, Freiburg, Germany). As negative controls, some sections were incubated with PBS in the absence of the primary antibodies: no immunoreactivity was observed in this condition.

#### 4.6.5. Transmission Electron Microscopy (TEM): UA and LC Staining

Lung fragments (small blocks of about 1 mm^3^) were fixed for 4 h by immersion in ice-cold 1.5% glutaraldehyde (Polysciences, Inc., Warrington, PA, USA) buffered with 0.07 M cacodylate buffer (pH 7.4), containing 7% sucrose, followed by post-fixation in OsO_4_ (Sigma Chemical Co., St. Louis, MO, USA) in 0.1 M cacodylate buffer (pH 7.4) for 2 h at 4 °C, dehydrated in a graded series of ethanol and embedded in Epon 812. For light microscopy pre-examination, semithin sections (1 micrometer thick) were stained with 1% borated methylene blue. For electron microscopy, ultrathin sections (about 600 Å thick) were cut from the blocks, mounted on uncoated 200-mesh-copper grids, and doubly stained with saturated uranyl acetate in 50% acetone and Reynold’s lead citrate solution. The specimens were examined with a Zeiss EM 300 electron microscope (Carl Zeiss S.p.A., Milan, Italy) operating at 80 kV.

#### 4.6.6. Semiquantitative Lung Lesion Analysis

A scoring system was utilized to evaluate the pulmonary histopathology regarding the extent of tumor progression, parenchyma and metastasis morphology and various tissue damages using conventional brightfield microscopy according to a semiquantitative scale ranging from absent/undetectable (−) to maximum (++++). Specifically, 20 sections per *n* = 3 animals were analyzed, examining 5 microscopic fields in each section for each mouse per time/condition.

The localization and degree of lesions were recorded and graded as follows: (−) absent/undetectable lesions; (+) mild injury; (++) moderate damage; (+++) severe alteration; (++++) complete tissue devastation. Specifically, the following alterations were recorded: (i) alveolar structure alteration, (ii) bronchiolar epithelial cells desquamation, (iii) hemorrhagic foci incidence, (iv) number of pulmonary metastases. Peculiar primary tumor cancer features were recorded (Table 1). Concerning the metastases, we also considered (i) localization in the width of the lung and (ii) density and metastatic tissue organization (columnar vs. nodular).

#### 4.6.7. Histochemical and Immunohistochemical Evaluations

For each selected marker, six slides (about 30 sections) per animal were analyzed. Pulmonary specimens with different immunolabelling extent were considered in all experimental groups. The figures show the most representative changes for each immunohistochemical reaction.

Histochemical and immunohistochemical labeling extent was evaluated on acquired digitized section images under exposure time avoiding any pixel saturation effect. The labeling intensity was measured utilizing densitometric analysis (Image-J 1.46p; NIH, Bethesda, MA, USA). The mask shape was adjusted depending on the spatial distribution of the lung specimens under measurement; the labeling was measured as the mean intensity value over the area.

The immunocytochemical intensity, indicated as optical density (OD), was evaluated in 30 cells/section per six slides/animal. Results were recorded on Microsoft Office Excel spreadsheets. OD was expressed as the product of OD value and immunopositive cell density (*10^3^).

The following further measurements were performed: (i) Picrosirius Red–positive bronchiolar surface in mm^2^/whole bronchiolar area in mm^2^ and (ii) immunopositive cells density count (number of immunopositive cells/area in mm^2^).

Concerning the metastases, both density (i.e., metastases number/surface area in mm^2^) and area (mean area in mm^2^/animal) were measured.

### 4.7. Statistics

Data were expressed as means ± standard error of the mean (SEM). The statistical analysis for Picrosirius Red staining was carried out using one-way ANOVA followed by Bonferroni’s post-hoc test. A Kruskal–Wallis non-parametric analysis of the semiquantitative histopathological data was performed, followed by Dunn’s post-hoc test. Concerning immunohistochemistry of the lung parenchyma, the statistical differences among all experimental groups, i.e., healthy controls, M. U-care–treated, and untreated mice, were measured by one-way ANOVA followed by Bonferroni’s post-hoc test. Differently, regarding the immunostainings of metastatic tissue, the statistical differences between M. U-care–treated and untreated mice were evaluated using an unpaired Student’s t-test.

Two-way ANOVA and Bonferroni post-hoc tests were performed to compare the different groups regarding behavioral analysis. The differences were considered statistically significant for *p* < 0.05 (*), *p* < 0.01 (**), and *p* < 0.001 (***).

Statistical analyses were performed by using GraphPad Prism 7.0 (GraphPad Software Inc., La Jolla, CA, USA) and R software.

## 5. Conclusions

In conclusion, our data revealed that the medicinal mushrooms blend supplementation, starting before 4T1 cells injection and lasting until the end of the experiment, elicited a dramatic decrease of pulmonary metastases density. Additionally, Micotherapy U-care triggered a significant decline of both inflammatory and oxidative stress pathways as evidenced by the reduction of IL-6, NOS2, and COX2 expression pattern. We hypothesize that the measured metastases decrease can be ascribable either to direct Micotherapy U-care anti-cancer effect son lung cells or to secondary/indirect impacts of the medicinal mushroom blend on systemic inflammation and immunomodulation. We cannot exclude that both mechanisms may contribute to the observed striking effects. To properly address this dilemma, our still ongoing studies are investigating whether and how Micotherapy U-care may affect the apoptotic pathway. Preliminary data on p53 and Bcl2, whose critical role in human breast cancer is well known, seem to demonstrate an inverse expression trend of these two proteins, being the p53 overexpressed in Micotherapy U-care–treated mice. If confirmed and corroborated by further experiments, these data might support a possible role of p53 in down-regulating Bcl-2, possibly explaining the apoptosis induction by wild-type p53.

Taken together, these findings corroborate the use of Micotherapy U-care blend as a novel strategy to be used in the field of integrative oncology to improve the patient quality of life and reduce adverse side effects due to conventional cancer treatments. Thus, although further investigations are necessary to translate these experimental findings to clinical setting, turning them into new clinical therapeutic protocols, the present study remarkably supports the valuable potential of medicinal mushrooms extracts, being a natural source of novel drugs, as adjuvant therapy in the critical management of TNBC.

## Figures and Tables

**Table 1:** Semiquantitative scoring of lung lesions in healthy control, untreated, and M. U-care–treated mice.

	Healthy Control	Untreated	M. U-care Treated	*p* Value
Alveolar structure alteration	−	+++	++	*
Haemorrhagic foci	−	++	++	ns
Bronchiolar desquamation	−	++	+	*
Number of pulmonary metastases	−	++	+	*

Degree scale: from absent/undetectable (−) to maximal (++++). *p* values calculated by Kruskal–Wallis followed by Dunn’s post hoc test: (*) <0.05; ns = not statistically significant.

**Table 2:** Details on Micotherapy U-care supplement composition.

Fungal Species	ID Code	Weight % Used in Blend	Part Used
*Agaricus blazei*	7700	20%	Sporophores
*Cordyceps sinensis*	Cm2	20%	Sporophores and mycelia
*Ganoderma lucidum*	Gač	20%	Sporophores
*Grifola frondosa*	Gf3	20%	Sporophores
*Lentinula edodes*	Le.ed.1	20%	Sporophores

**Table 3:** Nutrient composition of Micotherapy U-care (supplied by A. V. D. Reform s.r.l., Noceto, Parma, Italy).

Components	Per Capsule (mg)
*Ganoderma lucidum*	150 mg
*Grifola frondosa*	150 mg
*Agaricus blazei*	150 mg
*Cordyceps sinensis*	150 mg
*Lentinula edodes*	150 mg
Titled in polysaccharides (α plus β)	>30%
1,3-1,6 Beta-glucans	>15%

**Table 4:** Primary/secondary antibodies and respective dilution used for immunocytochemical experimental procedures.

	Antigen	Immunogen	Manufacturer, Species, Mono-polyclonal, cat./lot. No., RRID	Diluted Used
**Primary antibodies**	Anti-Interleukin-6 (M-19)	Purified antibody raised against a peptide mapping at the C-terminus of IL-6 of mouse origin	Santa Cruz Biotechnology (Santa Cruz, CA, USA), Goat polyclonal IgG, Cat# sc-1265, RRID: AB_2127470	1:100
	Anti-Transforming Growth Factor β1 (V)	Purified antibody raised against a peptide mapping at the C-terminus of TGF-β1 of human origin	Santa Cruz Biotechnology (Santa Cruz, CA, USA), Rabbit polyclonal IgG, Cat# sc-146, RRID: AB_632486	1:100
	Anti-Superoxide Dismutase-1 (FL-154)	Purified antibody raised against amino acids 1–154 representing full length SOD1 of human origin	Santa Cruz Biotechnology (Santa Cruz, CA, USA), Rabbit polyclonal IgG, Cat# sc-11407, RRID: AB_2193779	1:100
	Anti-Nitric Oxide Synthases-2 (M19)	Purified antibody raised against a peptide mapping at the C-terminus of NOS2 of mouse origin	Santa Cruz Biotechnology (Santa Cruz, CA, USA), Rabbit polyclonal IgG, Cat# sc-650, RRID: AB_631831	1:100
	Anti-Cyclooxygenase-2(M-19)	Purified antibody raised against a peptide mapping at the C-terminus of COX2 of mouse origin	Santa Cruz Biotechnology (Santa Cruz, CA, USA), Goat polyclonal IgG, Cat# sc-1747, RRID: AB_2084976	1:100
**Secondary antibodies**	Biotinylated goat anti-rabbit IgG	Gamma immunoglobulin	Vector Laboratories (Burlingame, CA, USA), Goat, lot# PK-6101, RRID: AB_2336820	1:200
	Biotinylated rabbit anti-goat IgG	Gamma immunoglobulin	Vector Laboratories (Burlingame, CA, USA), Rabbit, Cat# PK-6105, RRID: AB_2336824	1:200

## References

[1] Bray F., Ferlay J., Soerjomataram I., Siegel R.L., Torre L.A., Jemal A. (2018). Global cancer statistics 2018: GLOBOCAN estimates of incidence and mortality worldwide for 36 cancers in 185 countries. Ca Cancer J. Clin..

[2] WHO: Breast cancer. http://www.who.int/cancer/prevention/diagnosis-screening/breast-cancer/en/.

[3] Cardoso F., Harbeck N., Fallowfield L., Kyriakides S., Senkus E., ESMO Guidelines Working Group (2012). Locally recurrent or metastatic breast cancer: ESMO Clinical Practice Guidelines for diagnosis, treatment and follow-up. Ann. Oncol..

[4] Smith R.A., Duffy S.W., Tabár L. (2012). Breast cancer screening: The evolving evidence. Oncology.

[5] Yadav B.S., Chanana P., Jhamb S. (2015). Biomarkers in triple negative breast cancer: A review. World J. Clin. Oncol..

[6] Shao F., Sun H., Deng C.X. (2017). Potential therapeutic targets of triple-negative breast cancer based on its intrinsic subtype. Oncotarget.

[7] Yao Y., Chu Y., Xu B., Hu Q., Song Q. (2019). Risk factors for distant metastasis of patients with primary triple-negative breast cancer. Biosci. Rep..

[8] Wasser S.P. (2017). Medicinal Mushrooms in Human Clinical Studies. Part I. Anticancer, Oncoimmunological, and Immunomodulatory Activities: A Review. Int. J. Med. Mushrooms.

[9] Rossi P., Difrancia R., Quagliariello V., Savino E., Tralongo P., Randazzo C.L., Berretta M. (2018). B-glucans from *Grifola frondosa* and *Ganoderma lucidum* in breast cancer: An example of complementary and integrative medicine. Oncotarget.

[10] Blagodatski A., Yatsunskaya M., Mikhailova V., Tiasto V., Kagansky A., Katanaev V.L. (2018). Medicinal mushrooms as an attractive new source of natural compounds for future cancer therapy. Oncotarget.

[11] Jiang J., Sliva D. (2010). Novel medicinal mushroom blend suppresses growth and invasiveness of human breast cancer cells. Int. J. Oncol..

[12] Alonso E.N., Ferronato M.J., Fermento M.E., Gandini N.A., Romero A.L., Guevara J.A., Facchinetti M.M., Curino A.C. (2018). Antitumoural and antimetastatic activity of Maitake D-Fraction in triple-negative breast cancer cells. Oncotarget.

[13] Cai H., Li J., Gu B., Xiao Y., Chen R., Liu X., Xie X., Cao L. (2018). Extracts of *Cordyceps sinensis* inhibit breast cancer cell metastasis via down-regulation of metastasis-related cytokines expression. J. Ethnopharmacol..

[14] Mizuno T.K. (1995). Kawariharatake, *Agaricus blazei*Murrill medicinal and dietary effects. Food Rev. Int..

[15] Firenzuoli F., Gori L., Lombardo G. (2008). The Medicinal Mushroom *Agaricus blazei* Murrill: Review of Literature and Pharmaco-Toxicological Problems. Evid. Based Complement. Altern. Med..

[16] Da Silva de Souza A.C., Correa V.G., Goncalves G.d.A., Soares A.A., Bracht A., Peralta R.M. (2017). *Agaricus blazei* Bioactive Compounds and their Effects on Human Health: Benefits and Controversies. Curr. Pharm. Des..

[17] Kim Y.S., Kim B.H., Kim G.S., Jang J.S., Kim S.Y., Choi B.D., Kim J.O., Ha Y.L. (2014). Anti-carcinogenic actions of glycoprotein conjugated with isoflavones from submerged-liquid culture of *Agaricus blazei* mycelia through reciprocal expression of Bcl-2 and Bax proteins. J. Biomed. Res..

[18] De Sá-Nakanishi A.B., Soares A.A., de Oliveira A.L., Comar J.F., Peralta R.M., Bracht A. (2014). Effects of treating old rats with an aqueous *Agaricus blazei* extract on oxidative and functional parameters of the brain tissue and brain mitochondria. Oxid. Med. Cell Longev..

[19] Ohno S., Sumiyoshi Y., Hashine K., Shirato A., Kyo S., Inoue M. (2011). Phase I Clinical Study of the Dietary Supplement, *Agaricus blazei* Murill, in Cancer Patients in Remission. Evid. Based Complement. Altern. Med..

[20] Ahn W.S., Kim D.J., Chae G.T., Lee J.M., Bae S.M., Sin J.I., Kim Y.W., Namkoong S.E., Lee I.P. (2004). Natural killer cell activity and quality of life were improved by consumption of a mushroom extract, *Agaricus blazei* Murill Kyowa, in gynecological cancer patients undergoing chemotherapy. Int. J. Gynecol. Cancer.

[21] Jordan J.L., Nowak A., Lee T.D.G. (2010). Activation of innate immunity to reduce lung metastases in breast cancer. Cancer Immunol. Immunother..

[22] Wang J., Nie S., Cui S.W., Wang Z., Phillips A.O., Phillips G.O., Li Y., Xie M. (2017). Structural characterization and immunostimulatory activity of a glucan from natural *Cordyceps sinensis*. Food Hydrocol..

[23] Li S.P., Li P., Dong T.T., Tsim K.W. (2001). Anti-oxidation activity of different types of natural *Cordyceps sinensis* and cultured *Cordyceps mycelia*. Phytomedicine.

[24] Wu T.S., Shi L.S., Kuo S.C. (2001). Cytotoxicity of *Ganoderma lucidum* triterpenes. J. Nat. Prod..

[25] Jiang J., Slivova V., Harvey K., Valachovicova T., Sliva D. (2004). *Ganoderma lucidum* suppresses growth of breast cancer cells through the inhibition of Akt/NF-kappaB signaling. Nutr. Cancer.

[26] Sliva D., Sedlak M., Slivova V., Valachovicova T., Lloyd F.P., Ho N.W.Y. (2003). Biologic activity of spores and dried powder from *Ganoderma lucidum* for the inhibition of highly invasive human breast and prostate cancer cells. J. Altern. Complement. Med..

[27] Zhao H., Zhang Q., Zhao L., Huang X., Wang J., Kang X. (2012). Spore Powder of *Ganoderma lucidum* Improves Cancer-Related Fatigue in Breast Cancer Patients Undergoing Endocrine Therapy: A Pilot Clinical Trial. Evid. Based Complement. Altern. Med..

[28] Jin X., Ruiz Beguerie J., Sze D.M.Y., Chan G.C.F. (2016). *Ganoderma lucidum* (*Reishi mushroom*) for cancer treatment. Cochrane Database Syst. Rev..

[29] Soares R., Meireles M., Rocha A., Pirraco A., Obiol D., Alonso E.N., Joos G., Balogh G. (2011). Maitake (D fraction) mushroom extract induces apoptosis in breast cancer cells by BAK-1 gene activation. J. Med. Food.

[30] Alonso E.N., Orozco M., Nieto A.E., Balogh G.A. (2013). Genes Related to Suppression of Malignant Phenotype Induced by Maitake D-Fraction in Breast Cancer Cells. J. Med. Food.

[31] Said T.K., Moraes R.C., Singh U., Kittrell F.S., Medina D. (2001). Cyclin-dependent kinase (cdk) inhibitors/cdk4/cdk2 complexes in early stages of mouse mammary preneoplasia. Cell Growth Differ..

[32] Van ’t Veer L.J., Dai H., van de Vijver M.J., He Y.D., Hart A.A.M., Mao M., Peterse H.L., van der Kooy K., Marton M.J., Witteveen A.T. (2002). Gene expression profiling predicts clinical outcome of breast cancer. Nature.

[33] Doughtery E.R., Jianping H., Bittner M.L. (2007). Validation of Computational Methods in Genomics. Curr. Genom..

[34] Deng G., Lin H., Seidman A., Fornier M., D’Andrea G., Wesa K., Yeung S., Cunningham-Rundles S., Vickers A.J., Cassileth B. (2009). A phase I/II trial of a polysaccharide extract from *Grifola frondosa* (*Maitake mushroom*) in breast cancer patients: Immunological effects. J. Cancer Res. Clin. Oncol..

[35] Fang N., Li Q., Yu S., Zhang J., He L., Ronis M.J.J., Badger T.M. (2006). Inhibition of growth and induction of apoptosis in human cancer cell lines by an ethyl acetate fraction from shiitake mushrooms. J. Altern. Complement. Med..

[36] Ren Z., Li J., Song X., Zhang J., Wang W., Wang X., Gao Z., Jing H., Li S., Jia L. (2018). The regulation of inflammation and oxidative status against lung injury of residue polysaccharides by *Lentinula edodes*. Int. J. Biol. Macromol..

[37] Suzuki N., Takimoto Y., Suzuki R., Arai T., Uebaba K., Nakai M., Strong J.M., Tokuda H. (2013). Efficacy of oral administration of *Lentinula eododes* mycelia extract for breast cancer patients undergoing postoperative hormone therapy. Asian Pac. J. Cancer Prev..

[38] Nagashima Y., Maeda N., Yamamoto S., Yoshino S., Oka M. (2013). Evaluation of host quality of life and immune function in breast cancer patients treated with combination of adjuvant chemotherapy and oral administration of *Lentinula edodes* mycelia extract. Onco Targets Ther..

[39] Nagashima Y., Yoshino S., Yamamoto S., Maeda N., Azumi T., Komoike Y., Okuno K., Iwasa T., Tsurutani J., Nakagawa K. (2017). *Lentinula edodes* mycelia extract plus adjuvant chmotherapy for breast cancer patients: Results of a randomized study on host quality of life and immune function improvement. Mol. Clin. Oncol..

[40] Segnani C., Ippolito C., Antonioli L., Pellegrini C., Blandizzi C., Dolfi A., Bernardini N. (2015). Histochemical Detection of Collagen Fibers by Sirius Red/Fast Green Is More Sensitive than van Gieson or Sirius Red Alone in Normal and Inflamed Rat Colon. PLoS ONE.

[41] Pienta K.J., McGregor N., Axelrod R., Axelrod D.E. (2008). Ecological therapy for cancer: Defining tumours using an ecosystem paradigm suggests new opportunities for novel cancer treatments. Transl. Oncol..

[42] Song P., Zechner C., Hernandez G., Cánovas J., Xie Y., Sondhi V., Wagner M., Stadlbauer V., Horvath A., Leber B. (2018). The Hormone FGF21 Stimulates Water Drinking in Response to Ketogenic Diet and Alcohol. Cell Metab..

[43] Kim K.H., Lee M.S. (2014). FGF21 as a stress hormone: The roles of FGF21 in stress adaptation and the treatment of metabolic diseases. Diabetes Metab. J..

[44] Martínez-Garza U., Torres-Oteros D., Yarritu-Gallego A., Marrero P.F., Haro D., Relat J. (2019). Fibroblast Growth Factor 21 and the Adaptive Response to Nutritional Challenges. Int. J. Mol. Sci..

[45] Fischer A.H., Jacobson K.A., Rose J., Zeller R. (2008). Hematoxylin and eosin staining of tissue and cell sections. CSH Protoc..

[46] Kenny P.A., Lee G.Y., Myers C.A., Neve R.M., Semeiks J.R., Spellman P.T., Lorenz K., Lee E.H., Barcellos-Hoff M.H., Petersen O.W. (2007). The morphologies of breast cancer cell lines in three-dimensional assays correlate with their profiles of gene expression. Mol. Oncol..

[47] Krakhmal N.V., Zavyalova M.V., Denisov E.V., Vtorushin S.V., Perelmuter V.M. (2015). Cancer Invasion: Patterns and Mechanisms. Acta Nat..

[48] Hanahan D., Weinberg R.A. (2011). Hallmarks of cancer: The next generation. Cell.

[49] Liou G.Y., Storz P. (2010). Reactive oxygen species in cancer. Free Radic. Res..

[50] Fisher D.T., Appenheimer M.M., Evans S.S. (2014). The two faces of IL-6 in the tumour microenvironment. Semin. Immunol..

[51] Liubomirski Y., Lerrer S., Meshel T., Rubinstein-Achiasaf L., Morein D., Wiemann S., Körner C., Ben-Baruch A. (2019). Tumour-Stroma-Inflammation Networks Promote Pro-metastatic Chemokines and Aggressiveness Characteristics in Triple-Negative Breast Cancer. Front. Immunol..

[52] Garcia-Tuñón I., Ricote M., Ruiz A., Fraile B., Paniagua R., Royuela M. (2005). IL-6, its receptors and its relationship with bcl-2 and bax proteins in infiltrating and in situ human breast carcinoma. Histopathology.

[53] Coussens L.M., Zitvogel L., Palucka A.K. (2013). Neutralizing tumour-promoting chronic inflammation: A magic bullet?. Science.

[54] Mantovani A., Allavena P., Sica A., Balkwill F. (2008). Cancer-related inflammation. Nature.

[55] Grivennikov S.I., Greten F.R., Karin M. (2010). Immunity, inflammation, and cancer. Cell.

[56] Barbieri A., Quagliariello V., Del Vecchio V., Falco M., Luciano A., Amruthraj N.J., Nasti G., Ottaiano A., Berretta M., Iaffaioli R.V. (2017). Anticancer and Anti-Inflammatory Properties of *Ganoderma lucidum* Extract Effects on Melanoma and Triple-Negative Breast Cancer Treatment. Nutrients.

[57] Joshi A., Cao D. (2010). TGF-beta signaling, tumour microenvironment and tumour progression: The butterfly effect. Front. Biosci..

[58] Papageorgis P., Stylianopoulos T. (2015). Role of TGFβ in regulation of the tumour microenvironment and drug delivery (review). Int. J. Oncol..

[59] Moore-Smith L.D., Isayeva T., Lee J.H., Frost A., Ponnazhagan S. (2017). Silencing of TGF-β1 in tumour cells impacts MMP-9 in tumour microenvironment. Sci. Rep..

[60] Massagué J., Gomis R.R. (2006). The logic of TGFbeta signaling. FEBS Lett..

[61] Prasad S., Gupta S.C., Tyagi A.K. (2017). Reactive oxygen species (ROS) and cancer: Role of antioxidative nutraceuticals. Cancer Lett..

[62] Kumari S., Badana A.K., Malla R. (2018). Reactive Oxygen Species: A Key Constituent in Cancer Survival. Biomark Insights.

[63] Galadari S., Rahman A., Pallichankandy S., Thayyullathil F. (2017). Reactive oxygen species and cancer paradox: To promote or to suppress?. Free Radic. Biol. Med..

[64] Gu H., Huang T., Shen Y., Liu Y., Zhou F., Jin Y., Sattar H., Wei Y. (2018). Reactive Oxygen Species-Mediated Tumour Microenvironment Transformation: The Mechanism of Radioresistant Gastric Cancer. Oxid. Med. Cell Longev..

[65] Kepinska M., Kizek R., Milnerowicz H. (2018). Metallothionein and Superoxide Dismutase-Antioxidative Protein Status in Fullerene-Doxorubicin Delivery to MCF-7 Human Breast Cancer Cells. Int. J. Mol. Sci..

[66] Basudhar D., Bharadwaj G., Somasundaram V., Cheng R.Y.S., Ridnour L.A., Fujita M., Lockett S.J., Anderson S.K., McVicar D.W., Wink D.A. (2019). Understanding the tumour micro-environment communication network from an NOS2/COX2 perspective. Br. J. Pharm..

[67] Esbona K., Yi Y., Saha S., Yu M., Van Doorn R.R., Conklin M.W., Graham D.S., Wisinski K.B., Ponik S.M., Eliceiri K.W. (2018). The Presence of Cyclooxygenase 2, Tumour-Associated Macrophages, and Collagen Alignment as Prognostic Markers for Invasive Breast Carcinoma Patients. Am. J. Pathol..

[68] Brüne B., Courtial N., Dehne N., Syed S.N., Weigert A. (2017). Macrophage NOS2 in Tumour Leukocytes. Antioxid. Redox Signal..

[69] Thomas D.D., Wink D.A. (2017). NOS2 as an Emergent Player in Progression of Cancer. Antioxid. Redox Signal..

[70] Hashemi Goradel N., Najafi M., Salehi E., Farhood B., Mortezaee K. (2019). Cyclooxygenase-2 in cancer: A review. J. Cell Physiol..

[71] Yeh H.W., Lee S.S., Chang C.Y., Lang Y.D., Jou Y.S. (2019). A New Switch for TGFβ in Cancer. Cancer Res..

[72] Zhou K., Chen H., Lin J., Xu H., Wu H., Bao G., Li J., Deng X., Shui X., Gao W. (2019). FGF21 augments autophagy in random-pattern skin flaps via AMPK signalling pathways and improves tissue survival. Cell Death Dis..

[73] Su Z., Yang Z., Xu Y., Chen Y., Yu Q. (2015). Apoptosis, autophagy, necroptosis, and cancer metastasis. Mol. Cancer.

[74] Kim E.J., Choi M.R., Park H., Kim M., Hong J.E., Lee J.Y., Chun H.S., Lee K.W., Yoon Park J.H. (2011). Dietary fat increases solid tumor growth and metastasis of 4T1 murine mammary carcinoma cells and mortality in obesity-resistant BALB/c mice. Breast Cancer Res..

[75] Nigjeh S.E., Yeap S.K., Nordin N., Rahman H., Rosli R. (2019). In Vivo Anti-Tumor Effects of Citral on 4T1 Breast Cancer Cells via Induction of Apoptosis and Downregulation of Aldehyde Dehydrogenase Activity. Molecules.

[76] Fu Y., Guo F., Chen H., Lin Y., Fu X., Zhang H., Ding M. (2019). Core needle biopsy promotes lung metastasis of breast cancer: An experimental study. Mol. Clin. Oncol..

[77] Roda E., Bottone M.G., Biggiogera M., Milanesi G., Coccini T. (2019). Pulmonary and hepatic effects after low dose exposure to nanosilver: Early and long-lasting histological and ultrastructural alterations in rat. Toxicol. Rep..

[78] Brandalise F., Cesaroni V., Gregori A., Repetti M., Romano C., Orrù G., Botta L., Girometta C., Guglielminetti M.L., Savino E. (2017). Dietary Supplementation of *Hericium erinaceus* Increases Mossy Fiber-CA3 Hippocampal Neurotransmission and Recognition Memory in Wild-Type Mice. Evid. Based Complement. Altern. Med..

[79] Ratto D., Corana F., Mannucci B., Priori E.C., Cobelli F., Roda E., Ferrari B., Occhinegro A., Di Iorio C., De Luca F. (2019). *Hericium erinaceus* Improves Recognition Memory and Induces Hippocampal and Cerebellar Neurogenesis in Frail Mice during Aging. Nutrients.

[80] Weibel E.R. (1979). Morphometry of the human lung: The state of the art after two decades. Bull. Eur. Physiopathol. Respir..

[81] Avwioro G. (2011). Histochemical Uses of Haematoxylin-A Review. JPCS.

[82] Junqueira L.C., Bignolas G., Brentani R.R. (1979). Picrosirius staining plus polarization microscopy, a specific method for collagen detection in tissue sections. Histochem. J..

[83] Lattouf R., Younes R., Lutomski D., Naaman N., Godeau G., Senni K., Changotade S. (2014). Picrosirius red staining: A useful tool to appraise collagen networks in normal and pathological tissues. J. Histochem. Cytochem..

